# Malaria early warning tool: linking inter-annual climate and malaria variability in northern Guadalcanal, Solomon Islands

**DOI:** 10.1186/s12936-017-2120-5

**Published:** 2017-11-21

**Authors:** Jason Smith, Lloyd Tahani, Albino Bobogare, Hugo Bugoro, Francis Otto, George Fafale, David Hiriasa, Adna Kazazic, Grant Beard, Amanda Amjadali, Isabelle Jeanne

**Affiliations:** 1 Australian Bureau of Meteorology, 700 Collins St, Docklands, Melbourne, VIC 3008 Australia; 2 Solomon Islands Meteorological Service, Honiara, Capital Territory Solomon Islands; 3 National Vector Borne Disease Control Programme, Honiara, Capital Territory Solomon Islands; 4 Pacific Science Solutions, Suva, Fiji

**Keywords:** Malaria, Solomon Islands, Guadalcanal, Climate, Rainfall, Early warning, *Anopheles farauti*

## Abstract

**Background:**

Malaria control remains a significant challenge in the Solomon Islands. Despite progress made by local malaria control agencies over the past decade, case rates remain high in some areas of the country. Studies from around the world have confirmed important links between climate and malaria transmission. This study focuses on understanding the links between malaria and climate in Guadalcanal, Solomon Islands, with a view towards developing a climate-based monitoring and early warning for periods of enhanced malaria transmission.

**Methods:**

Climate records were sourced from the Solomon Islands meteorological service (SIMS) and historical malaria case records were sourced from the National Vector-Borne Disease Control Programme (NVBDCP). A declining trend in malaria cases over the last decade associated with improved malaria control was adjusted for. A stepwise regression was performed between climate variables and climate-associated malaria transmission (CMT) at different lag intervals to determine where significant relationships existed. The suitability of these results for use in a three-tiered categorical warning system was then assessed using a Mann–Whitney U test.

**Results:**

Of the climate variables considered, only rainfall had a consistently significant relationship with malaria in North Guadalcanal. Optimal lag intervals were determined for prediction using R^2^ skill scores. A highly significant negative correlation (R = − 0.86, R^2^ = 0.74, p < 0.05, n = 14) was found between October and December rainfall at Honiara and CMT in northern Guadalcanal for the subsequent January–June. This indicates that drier October–December periods are followed by higher malaria transmission periods in January–June. Cross-validation emphasized the suitability of this relationship for forecasting purposes ${\text{R}}^{2}{_{\text{LOOCV}}} = 0. 6 3$  as did Mann–Whitney U test results showing that rainfall below or above specific thresholds was significantly associated with above or below normal malaria transmission, respectively.

**Conclusion:**

This study demonstrated that rainfall provides the best predictor of malaria transmission in North Guadalcanal. This relationship is thought to be underpinned by the unique hydrological conditions in northern Guadalcanal which allow sandbars to form across the mouths of estuaries which act to develop or increase stagnant brackish marshes in low rainfall periods. These are ideal habitats for the main mosquito vector, *Anopheles farauti*. High rainfall accumulations result in the flushing of these habitats, reducing their viability. The results of this study are now being used as the basis of a malaria early warning system which has been jointly implemented by the SIMS, NVBDCP and the Australian Bureau of Meteorology.

## Background

### Malaria in the Solomon Islands

Malaria transmission is a significant problem in the western Pacific countries of the Solomon Islands, Vanuatu and Papua New Guinea, which lie on the eastern fringe of the global malaria endemic region. In recent years, there has been a renewed major focus on malaria eradication at the edges of this region to effectively shrink the worldwide malaria endemic region [1–4]. Under this strategy, the countries of the western Pacific, including the Solomon Islands and Vanuatu have become an obvious target for malaria elimination.

In the Solomon Islands malaria remains a significant cause of morbidity, particularly in young children. In the early 1990s, malaria incidence in the Solomon Islands was amongst the highest in the world outside of sub-Saharan Africa. In 1993, the Solomon Islands government began implementing a coordinated control programme administered at the provincial level and centred on the use of insecticide-treated bed nets, house spraying using DDT and community awareness programs [5]. However, with the precise composition of these programmes and their associated funding allocations decided at the provincial level, there was substantial variation in the effectiveness of control practices across the country.

In the early 2000s, ethnic violence led to significant disruptions in malaria control programmes in the Solomon Islands. By 2005, malaria incidence had risen to levels not seen since the mid-90s, particularly in the two heavily effected regions, Honiara Capital City Territory (HCC) and Guadalcanal Province. In 2007, the government of the Solomon Islands embarked on a new “National Malaria Strategic Plan, 2007–2016” with the aim of reducing national malaria incidence by over 75% as well as achieving malaria elimination in Temotu province by 2016 [6].

Since the implementation of this programme, infection rates have declined dramatically and, in 2011, the annual parasite incidence (API) across the Solomon Islands had dropped to just 49.1 cases per 1000 population, the lowest level since the 1970s [7]. The API is now below 100 in all provinces but significant spatial variability in malaria incidence remains. Relatively high malaria incidence persists in three provinces, Guadalcanal, Malaita and Central Provinces and HCC. Evidence from the last few years also suggests that progress has begun to slow, with annual case numbers reaching a plateau since 2011 [8, 9].

The relationship between climate and malaria has been established in a number of different malaria-affected regions of the world [10–12]. In particular, the effects of rainfall and temperature variation on vector breeding cycles and human malaria incidence rates are an area of ongoing research. A key finding of previous studies is the contrasting response of malaria incidence to rainfall in different regions of the world, and the sensitivity of this response to regional hydrological conditions. Research in some wet equatorial areas have shown a significant negative correlation between rainfall and malaria incidence, in South America, South–East Asia and the Pacific such as Sri Lanka [13], Columbia [14], Venezuela [15], French Guiana [16], New Guinea [17] and the Solomon Islands [18]. However, research in dry tropical and sub-tropical areas has typically shown a significant positive correlation with rainfall, such as South Africa [19], Botswana [20], Malawi [21], Ghana [22], Tanzania [23] and the East African Highlands [24]. Of particular interest is the existence of lagged relationships between climate drivers, rainfall and malaria which provide scope for the development of climate-based malaria early warning systems [20, 25–29].

The relationship between rainfall and malaria transmission is strongly influenced by local topography, land use, land cover including vegetation and hydrological features, along with the habitat preferences of local vector species. This means that extrapolation of specific results from one region to another is subject to significant uncertainty. Local observations are essential to understanding the relationship between climate and malaria in any particular region. Although a small number of studies have discussed or analysed the relationship between environment and malaria in the Solomon Islands, there has not yet been a comprehensive study to establish the predictability of malaria transmission based on climate [18, 30–32].

### Climate in the Solomon Islands

**Fig. 1:**
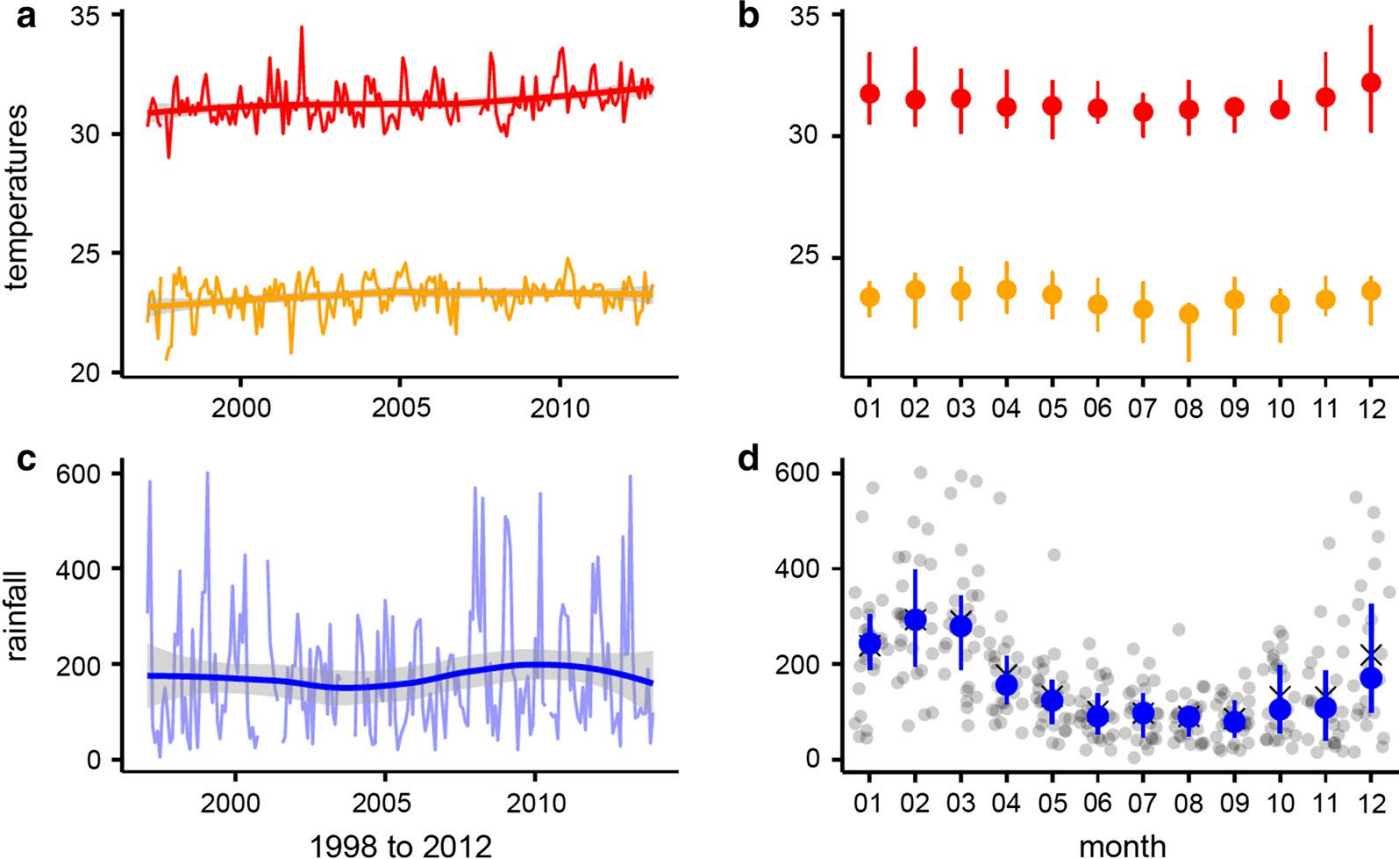
Monthly temperatures and rainfall, recorded in Honiara station from 1998 to 2012. **a**, **b** Monthly minimum (orange) and maximum (red) temperatures (°C) by year (**a**) and by month (**b**). **c**, **d** Monthly rainfall (mm) by year (**c**) and by month (**d**). Mean values for each month are denoted by x, the median by a circle and quartiles by vertical lines

The Solomon Islands is an archipelago of over 900 islands in the southwest Pacific, sitting within the western Pacific Warm Pool (see Annex for a glossary of climate terms). The Solomon Islands experiences a humid tropical climate with consistently high temperatures year round (Fig. 1a). Monthly maximum temperatures peak in December and January at around 32 °C while monthly minimum temperatures are at their lowest between August and October with values of around 23 °C (Fig. 1b). Monthly rainfall in this part of the country varies annually (Fig. 1c) and seasonally (Fig. 1d).

The tropical cyclone season in the Solomon Islands, resulting in flooding and wind damage, is between November and April with a large interannual variability. Due to the complicated topography of the Solomon Islands and its position near the confluence of the inter-tropical convergence zone (ITCZ) and the South Pacific convergence zone (SPCZ), rainfall across the country varies considerably. Most of the larger islands in the group, including Guadalcanal, are rugged and mountainous with the windward side experiencing higher annual rainfall and less seasonality. Locations on the leeward side of the islands typically experience lower annual rainfall and more distinct wet and dry seasons. The capital, Honiara, on the northwest (leeward) coast of Guadalcanal, receives an average annual rainfall of around 2200 mm with distinct wet (Nov–Apr) and dry (May–Oct) seasons [33]. Climate on the southeast (windward) coast of Guadalcanal is not continuously monitored, but sporadic historical observations indicate that this coast receives an average annual rainfall in excess of 4000 mm, with less distinct wet and dry seasons [34, 35].

Rainfall on Guadalcanal exhibits significant inter-annual variability due to the influence of the El-Niño southern oscillation (ENSO). The El Niño phase of ENSO tends to be hotter and drier whilst the La Niña phase tends to be cooler and wetter. The difference in rainfall during these phases is also significant, with Guadalcanal receiving almost double the amount of rainfall in wetter years relative to drier years [33]. It has also been observed that the lower rainfall associated with El Niño allows the formation and persistence of sand ridges at the mouths of permanent water courses in northern Guadalcanal, resulting in the enlargement of the stagnant, brackish marshes which are the preferred habitat of *Anopheles farauti* [30].

This study explores the relationship between the seasonal and annual variations in climate parameters such as rainfall, temperature, sea level and ENSO (SOI and NINO 3.4), and malaria transmission in Guadalcanal. The findings of this study will be used to assess the feasibility of implementing a climate-based malaria early warning system in the Solomon Islands that could assist local malaria control agencies with the implementation of targeted malaria control, reduction and awareness strategies.

## Methods

### Malaria data

The National Vector-Borne Disease Control Programme (NVBDCP) is responsible for malaria control and monitoring in the Solomon Islands and is charged with the collection and storage of malaria data. Likewise, the Solomon Islands meteorological service (SIMS) is responsible for climate monitoring and forecasting and is responsible for the collection and storage of climate data.

The NVBDCP provided monthly passive case detection (PCD) data aggregated at the provincial level for the period 1988–2013, providing 21 years of suitable data once gaps were taken into account. Sub-provincial data were only available from 1998 onwards, providing 14 years of suitable data at this spatial resolution. Data for a part of 1999 and to 2001 was unreliable and incomplete in both data sets, due to ethnic conflicts taking place at this time. Data collection changed and improved over years.

**Fig. 2:**
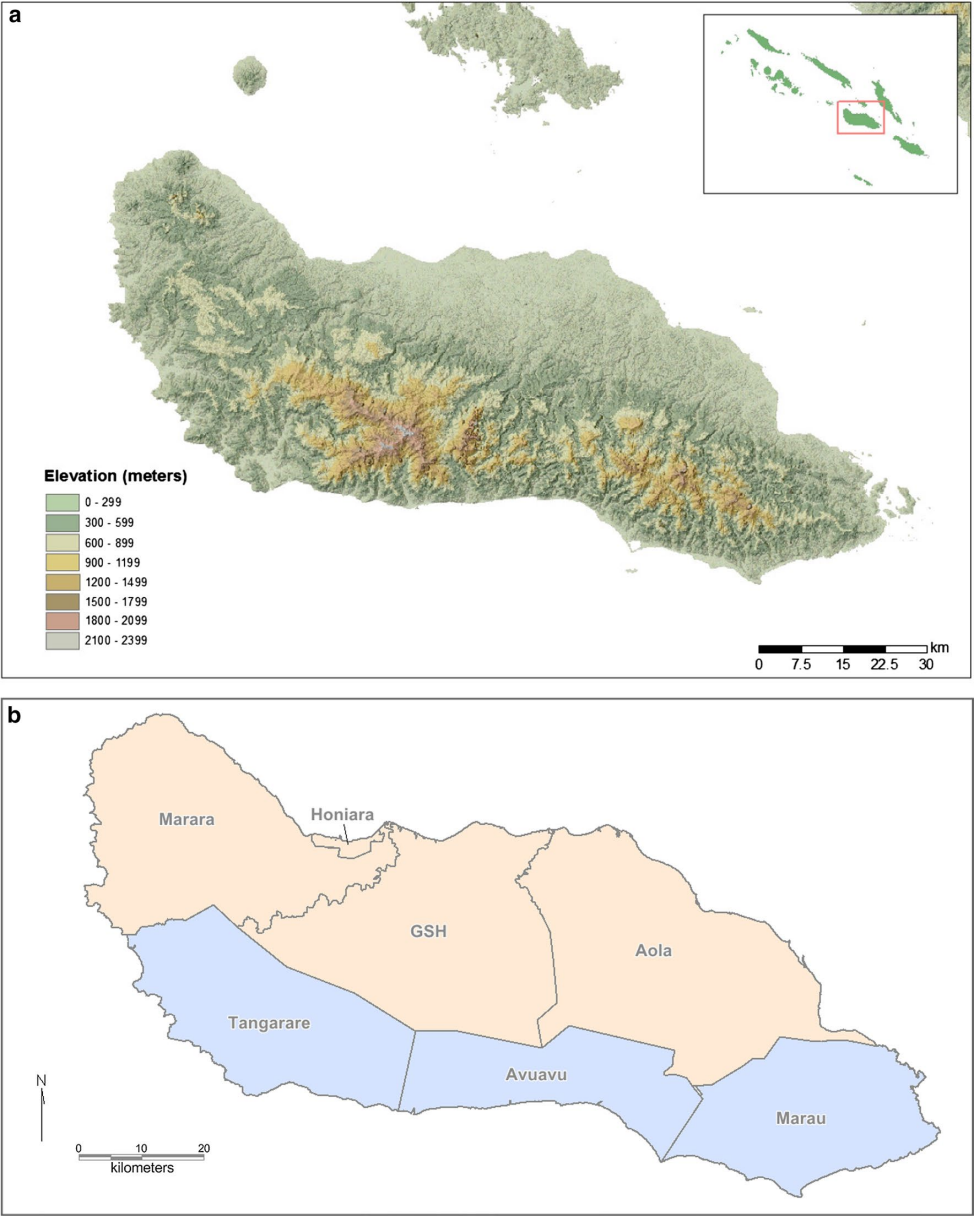
**a** Relief map of Guadalcanal, realised from ASTERGDEM USGS data (https://asterweb.jpl.nasa.gov/gdem.asp): due to the prevailing southeasterly trade winds, rainfall is higher on the south and southeast (windward) and lower in the north and northwest (leeward) side of the island. **b** Administrative health regions of Guadalcanal, including aggregated northern (yellow) and southern (blue) Guadalcanal regions used in this analysis

At sub-provincial level, Guadalcanal is administered as six health zones named Marara, GSH, Aola, Marau, Avuavu and Tangarare. This excludes the region surrounding Honiara which is separately administered as the Capital Territory of Solomon Islands. Guadalcanal can be divided into northern and southern regions by the highlands which bisect the island (Fig. 2a). Northern Guadalcanal is defined as Marara, GSH and Aola, while southern Guadalcanal is defined as Tangarare, Avuavu and Marau (Fig. 2b).

### Climate data

The SIMS provided monthly aggregated rainfall data for Vavaya Ridge (Honiara weather office) and Henderson Airport (11 km east of Honiara). Mean sea level (MSL) data were sourced from tidal measurement equipment in the Port of Honiara, jointly administered by SIMS and the Australian Bureau of meteorology’s Pacific sea level monitoring project (PSLM) [36]. Finally, the southern oscillation index (SOI) data, a measure of the pressure difference across the tropical south Pacific (Darwin, Australia and Tahiti, French Polynesia), and the definition of ENSO years were sourced from the Australian Bureau of Meteorology [37], while the United States National Oceanic and Atmospheric Administration (NOAA) [38] was used as a source of NINO3.4 data, which is a measure of SST anomalies in the central Pacific region bounded by 5°N–5°S and 170°W–120°W. These indices are used as indicators of the current ENSO phase which is the major driver of inter-annual rainfall and temperature variation in the Solomon Islands [33, 39].

### Population data

Northern Guadalcanal accounted for over 75% of all observed malaria cases in Guadalcanal between 2002 and 2013. Population data for Guadalcanal also indicated that the proportion of the population in the northern region has grown from 66% in 1998 to 79% in 2013. This information suggested that the relationship between climate and malaria shown at provincial level would likely be similar to the relationship identified for the northern region alone. This meant that the relationship between climate and malaria at the provincial level, where a longer more robust dataset exists, could be a useful surrogate for the relationship in northern Guadalcanal where only 14 years of data are available. Conversely, the preponderance of malaria cases in the north of the province meant that the provincial level data was unlikely to give us useful guidance about the relationship between climate and malaria in southern Guadalcanal.

### The three PCD datasets examined were

1. 1988–2013: Guadalcanal Cases (excl. 1989, 1991, 2000 and 2001).
2. 1998–2013: Northern Guadalcanal Cases (excl. 2000 and 2001).
3. 1998–2013: Southern Guadalcanal Cases (excl. 2000 and 2001).

Population data from the 1986, 1999 and 2009 national censuses were used to linearly interpolate annual population for Guadalcanal (1986–2009), northern Guadalcanal (1998–2009) and southern Guadalcanal (1998–2009). Population estimates from the Ministry of Health and Medical Services were used for the period 2010–2013. Malaria incidence (I) was defined as the number of malaria cases per 1000 population for a specified region. Not all years of data between 1988 and 2013 were available from the NVBDCP, with no monthly malaria incidence data available for 1990 and 1992. Examination of underlying health centre data also revealed a substantial degree of under-reporting during the years 1999–2001, a fact confirmed in discussions with provincial NVBDCP officers.

### Lagged statistical relationship between climate and malaria data

Preliminary analysis of the data was undertaken using a statistical climate tool called “seasonal climate outlooks in Pacific Island countries” (SCOPIC) [40]. Developed by the Australian Bureau of Meteorology with funding from the Department of Foreign Affairs, SCOPIC is specifically designed for exploring lagged statistical relationships between two variables [41, 42]. The software allows for the adjustment of the independent variable (predictor) period, dependent variable (predictand) period and the lag interval between the two variables.

Using SCOPIC, the relationships between the different climate and malaria time series were analysed in order to locate periods of the year where predictability of malaria incidence was significant. The strength of the relationship between two variables was assessed using the cross-validated R^2^. Lagged relationships of up to 6 months between climate and malaria were of particular interest in determining whether climate indices could be used to provide an early warning for changes in malaria transmission.

Ordinary least squares regression was performed between each of the available climate variables for the best early indicator of the malaria incidence variability. The lagged relationship between each of the available climate variables and malaria incidence was assessed in isolation.

### Adjustment for non-climate decadal trend in malaria transmission

**Fig. 3:**
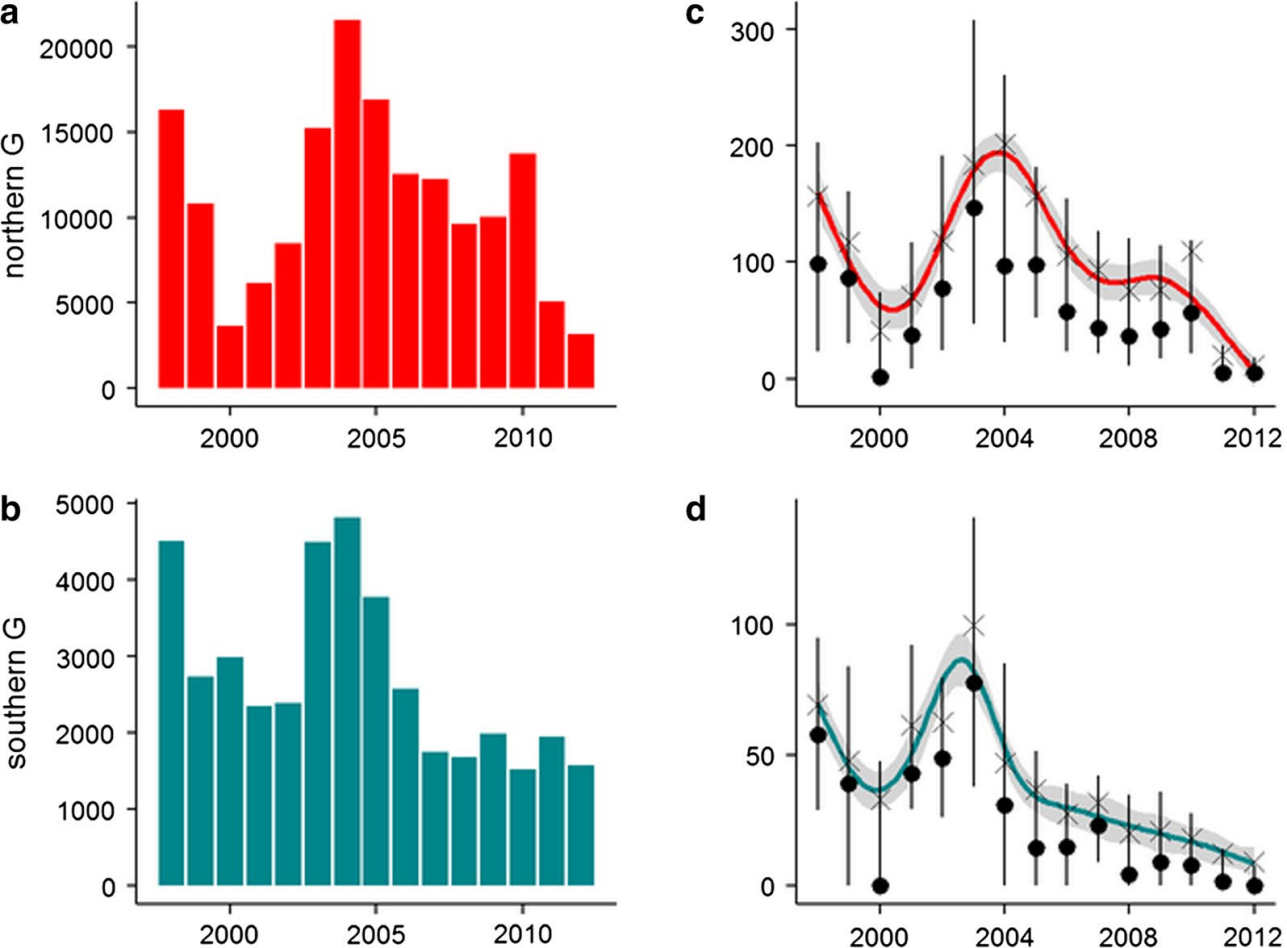
Malaria cases in Guadalcanal, 1998–2013. First row: **a**, **c**, northern Guadalcanal cases. Second row: **b**, **d**, southern Guadalcanal cases. **a**, **b** Recorded annual observed malaria cases. In 2000 and 2001, a few data were recorded due to political events. **c**, **d** Recorded monthly cases reported by health centre and summarised by year: mean (x), median (•) and quartiles from 0.25 to 0.75 (vertical line) and smooth line (Loess method)

A significant downward trend in annual malaria cases was apparent over the last decade (Fig. 3). This trend appeared to be associated with an increase in international malaria funding between 2003 and 2007 and the subsequent intensification of malaria control activities from the mid-2000s onwards [6, 7]. Adjustments were made to the time series in order to eliminate this non-climate related trend from 2005 onwards, although a lack of quantitative control data meant that the trend had to be estimated using a simple curve-fitting approach.

It was assumed that the intensification of control efforts could be modelled as a monotonic decline in cases over the last decade with inter-annual climate variability driving the variability around the downward trend. Linear and logarithmic functions were fitted over the last 10 years of data to estimate the downward trend in malaria cases since 2005 with the logarithmic function providing a more accurate fit to the observed data. The climate-associated malaria transmission, CMT, was defined to represent the time series which had been de-trended after 2005:

$${\text{CMT}} = {\text{Log}}_{\text{e}} \left( {\text{I}} \right) + {\text{C}}_{\text{trend}} ,$$

where C_trend_ = C_const_ × Log_e_(C_year_ + 1) and C_year_ = 0 for year ≤ 2005 and C_year_ = C_year − 1_ + 1 for year > 2005.

**Fig. 4:**
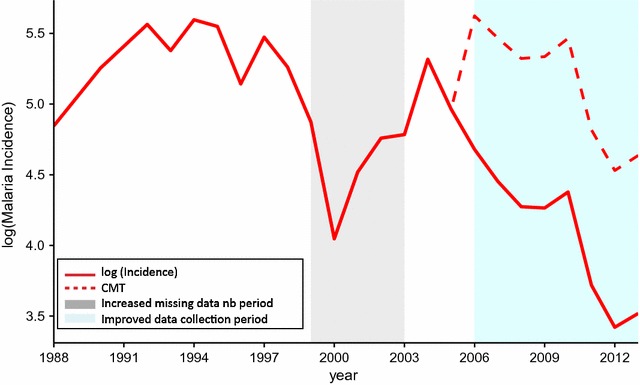
Adjusted malaria incidence. Comparison of natural logarithm of observed malaria incidence (solid) and logarithm of adjusted malaria incidence with late-period control trend removed (dashed) in Guadalcanal over the period 1988–2013

C_year_ takes into account the years in which the national control programme was greatly intensified. C_const_ is a multiplier which is scaled to fit the log curve associated with C_year_ to the trend in observed data (Fig. 4).

Subsequent statistical analyses were based on CMT, rather than the raw incidence data, as this variable better reflected the component of the malaria transmission associated with climate factors. The difference between CMT and log cases is shown in Fig. 4. CMT_NG_, CMT_SG_ and CMT_GP_ were defined as the climate-associated malaria transmission for northern Guadalcanal, southern Guadalcanal and Guadalcanal Province.

To assess the available climate variables, a forward backward stepwise method was used to build the regression model, with CMT using p < 0.05 as the criterion for inclusion (forward) and p > 0.1 as the criterion for omission (backward). Since rainfall was expected to have the strongest relationship to malaria transmission, a higher criterion for omission meant that the stepwise regression was more likely to retain other climate variables with smaller but possibly important effects on malaria transmission. Ninety-five percent confidence intervals for the regression coefficients were calculated using 1000 bootstrap samples [43].

The predictive power of the models was assessed by performing Leave-One-Out Cross-Validation (LOOCV) to calculate the Predictive Residual Error Sum of Squares (PRESS) and hence the cross-validated R^2^ $\left({\text{R}}^{2}{_{\text{LOOCV}}}\right)$ [44]. An excessive difference between R^2^ and ${\text{R}}^{2}{_{\text{LOOCV}}}$ may indicate that the model being analysed is not likely to be suitable for making predictions.

Some graphs and statistical analysis were updated using the R software version 3.4.0 [45] with the following packages: ggplot2 [46], tidyverse [47] and forecast [48].

### Threshold determination for operational use

In collaboration with the SIMS and NVBDCP, it was decided that a three-tier categorical warning system would be the most appropriate format for disseminating malaria alerts for the upcoming season. This meant that the relationships explored were transformed into three discrete warning levels: below normal, normal and above normal. In climate, three-category statistical forecasting systems are typically derived from the terciles of the historical climate dataset being used. Likewise, lower, middle and upper tercile years for malaria transmission were categorised as below normal, normal and above normal respectively. Rainfall thresholds were then calculated using the regression relationship between rainfall and malaria transmission in order to create a three-tiered categorical early warning system.

## Results

### Lagged relationship between climate and malaria

**Fig. 5:**
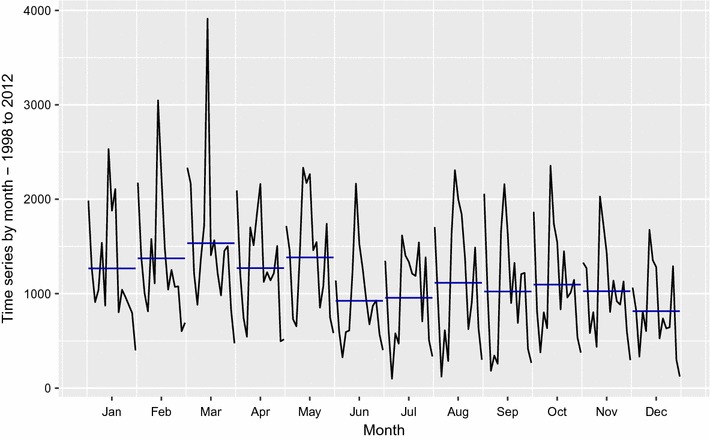
Inter annual variability of malaria cases in Guadalcanal, 1998–2012, separated by month. Each horizontal blue line represents the mean for each month over the years

The monthly mean malaria cases in Guadalcanal can be roughly divided into two main periods, January–June when the cases numbers are higher, peaking in March, and July–December with generally lower number cases (Fig. 5).

**Fig. 6:**
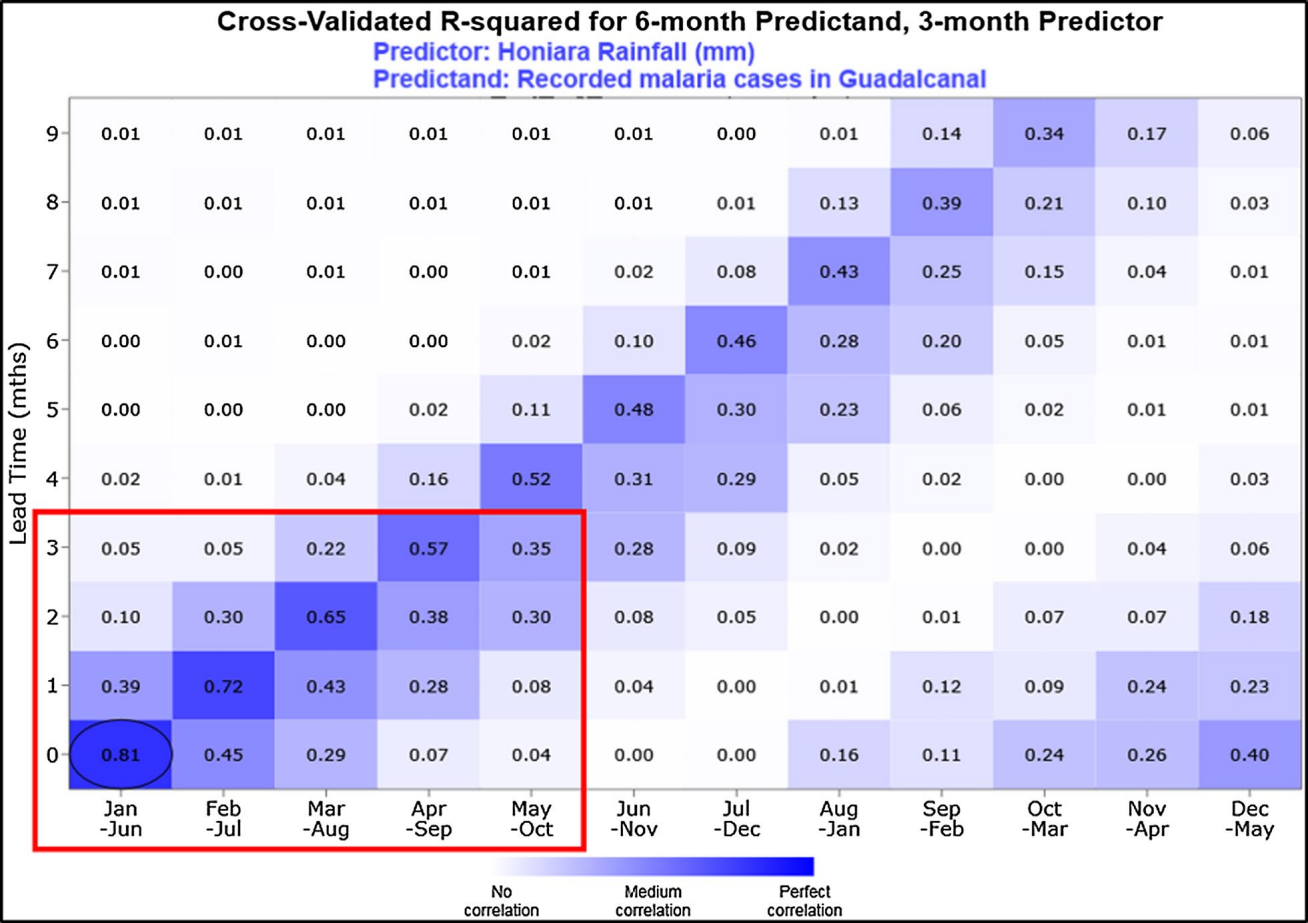
Forecast skill in northern Guadalcanal for 3 months predictor (Honiara rainfall) and 6 months predictand (CMT_GP_), from the 1988 to 2013 dataset. The above diagram shows that strong skill exists for predictions of climate-associated malaria transmission in the early part of the year with 0–2 months lead time

SCOPIC showed that the predictability of malaria incidence based on climate variables in the first half of the year was significantly better than in the second half, where little predictability existed. Focusing on the peak in the first half of the year, the variability in October–December (OND) rainfall appeared to be the best early indicator of the variability in January–June malaria incidence in the following year (Fig. 6).

**Fig. 7:**
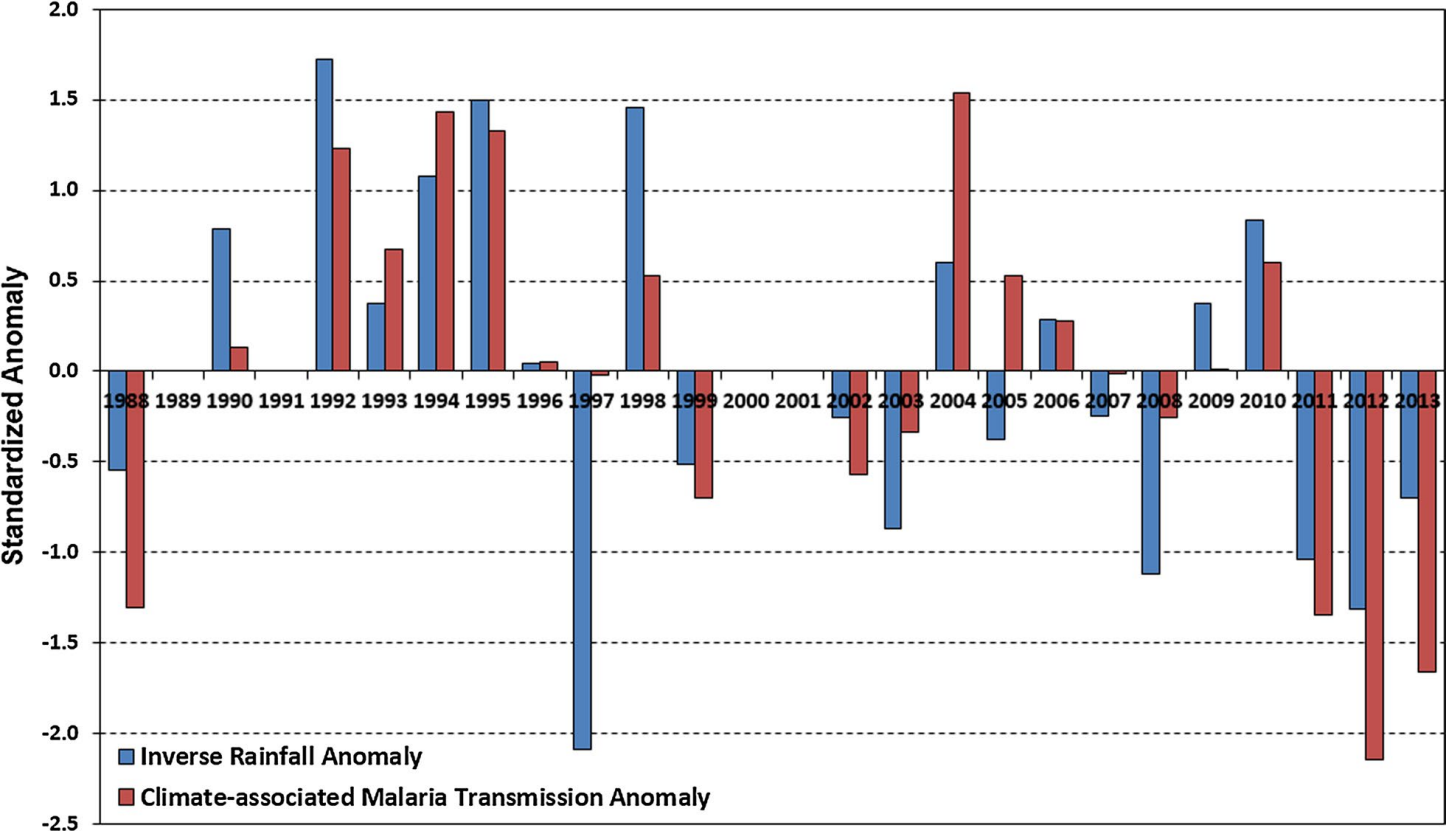
January–June climate-associated malaria transmission for Guadalcanal Province (CMT_GP_) plotted beside preceding October–December inverse rainfall anomaly at Vavaya Ridge rainfall gauge in Honiara. The close relationship can be seen where the negative standardized rainfall anomaly for OND is plotted against the January–June CMT_GP_ over the period 1988–2013. Above average rainfall preceded below average malaria transmission while below average rainfall preceded above average malaria transmission for most of the period data was available

A negative relationship was found to exist between rainfall and malaria transmission in Guadalcanal Province (Fig. 7). In particular, it shows that in all 11 years with a positive OND rainfall anomaly, only one negative Jan–Jun CMT_GP_ anomaly occurred (in 2005). On the other hand, all 11 years with a negative OND rainfall anomaly recorded a positive Jan–Jun CMT_GP_ anomaly. Apart from 2005, the only other year with a significant difference between modelled and observed transmission was in 2008. In this case the prediction and the observed transmission were both below normal but the difference in magnitude between the two was almost one standard deviation.

**Fig. 8:**
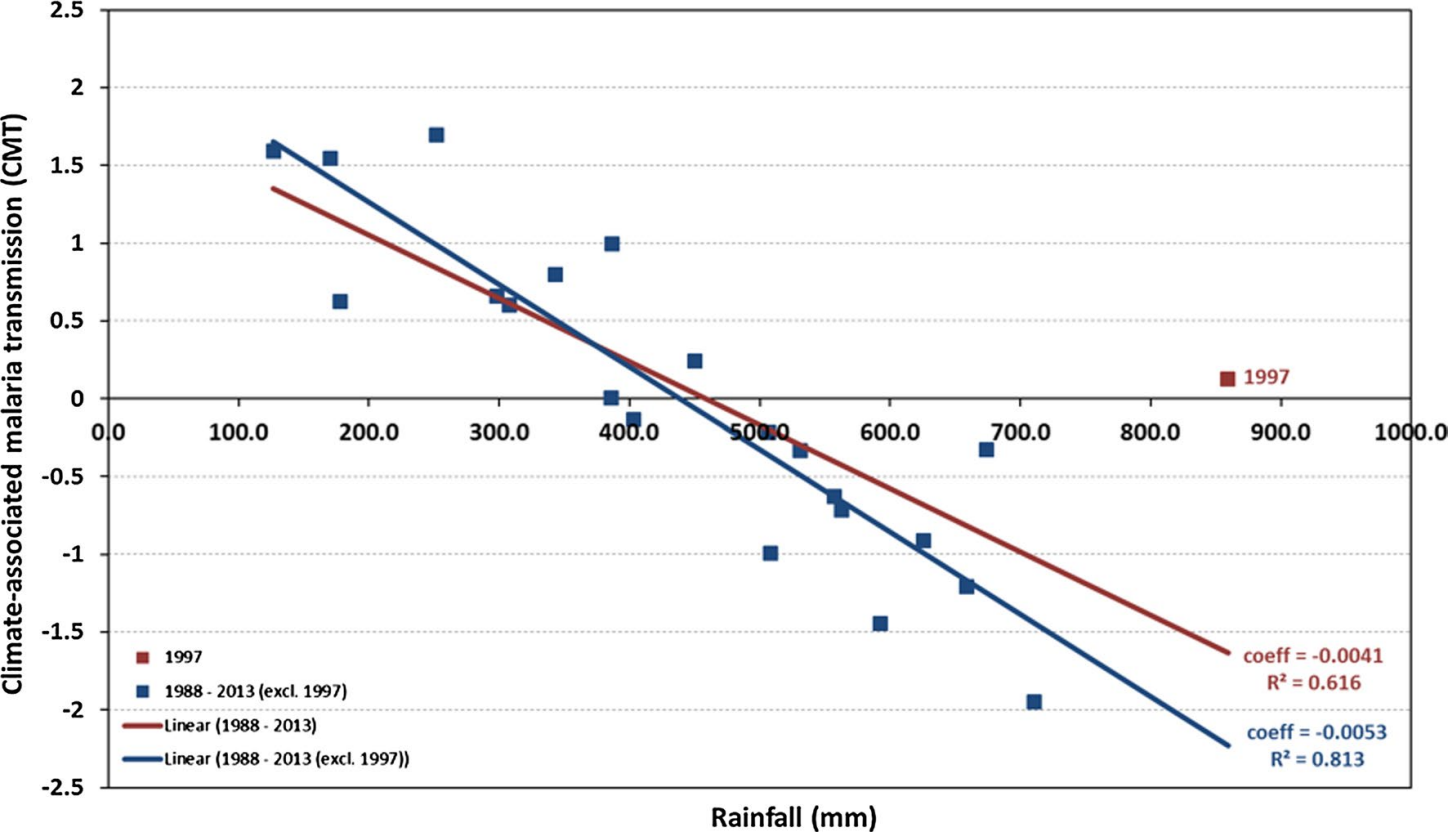
Comparison of fit and statistics for regression between January–June CMT_GP_ and October–December rainfall at Honiara with (red) and without (blue) outlier year 1997

Grubbs outlier test [49] revealed that the regression results for 1997 to be a significant outlier. The Cook’s distance [50] of the 1997 point also indicated a high leverage associated with the point suggesting a significant impact on the fit of the regression. The extent to which the 1997 data point was an outlier and its effect on the fit of the regression are shown in Fig. 8. A large OND negative rainfall anomaly was followed by a near average Jan-Jun CMT_GP_. In the last days of December 1996 a tropical cyclone resulted in heavy rainfall in Guadalcanal while conditions in the months preceding this were relatively dry. This may explain why 1997 malaria may not have been well predicted. Nevertheless, it was decided that this point would be discarded from all subsequent analysis for Guadalcanal Province.

The slope of the regression between CMT and rainfall is similar for both Guadalcanal Province and northern Guadalcanal (CMT_GP_ = 0.0053 and CMT_NG_ = 0.0055) but the intercept for CMT_NG_ is significantly higher owing to the 2000–2010 period being wetter than the 1990–2000 period (Tables 1 and 2).

**Table 1:** 21-year Guadalcanal

Variable	p value	Adjusted R^2^	Coefficient	Intercept
Rainfall	2.48E−8	0.80 [0.58, 0.90]	− 0.0053 [− 0.0062, − 0.0038]	2.272 [1.644, 2.799]
NINO3.4	0.061	0.13 [0, 0.42]	0.1276 [0.026, 0.257]	0.0 [− 0.418, 0.406]
SOI	0.006	0.30 [0.04, 0.60]	− 0.0195 [− 0.031, − 0.009]	− 0.0346 [− 0.397, 0.309]

Summary of linear regression results for data including 95% bootstrap confidence intervals in brackets (1000 bootstrap samples)

**Table 2:** 14-year northern Guadalcanal

Variable	p value	Adjusted R^2^	Coefficient	Intercept
Rainfall	7.42E−5	0.72 [0.27, 0.86]	− 0.0055 [− 0.0036, − 0.0076]	2.738 [1.844, 3.941]
MSL	0.033	0.27 [0, 0.67]	− 5.5236 [− 10.132, − 1.252]	3.683 [0.962, 7.113]
NINO3.4	0.036	0.26 [0, 0.62]	0.1497 [0.0092, 0.2803]	0.052 [− 0.337, 0.513]
SOI	0.079	0.17 [0, 0.62]	− 0.0146 [− 0.0329, 0.0017]	0.095 [− 0.338, 0.616]

Summary of linear regression results for data including 95% bootstrap confidence intervals in brackets (1000 bootstrap samples). Rainfall for the October–December period exhibited the strongest relationship with the climate-associated malaria transmission in the subsequent 6-month period for northern Guadalcanal (January–June)

Rainfall for the OND period exhibited the strongest relationship with the climate-associated malaria transmission in the subsequent 6-month period for both northern Guadalcanal and Guadalcanal Province. This relationship is suitable for predictions as emphasized by cross validation (R^2^ = 0.74 [0.32 − 0.88] 95% confidence interval, calculated using 1000 bootstrap samples and ${\text{R}}^{2}{_{\text{LOOCV}}} = 0. 6 3$) (Table 3).

**Table 3:** October to December rainfall and subsequent January to June malaria incidence

Region	Points	Pearson (r)	Spearman (ρ)	R^2^	${\text{R}}^{2}{_{\text{adj}}}$	${\text{R}}^{2}{_{\text{LOOCV}}}$
Guadalcanal (CMT_GP_)	21	− 0.90 [− 0.77, − 0.95]	− 0.90 [− 0.75, − 0.97]	0.81 [0.60, 0.91]	0.80 [0.58, 0.90]	0.77
Northern Guadalcanal (CMT_NG_)	14	− 0.86 [− 0.57, − 0.94]	− 0.84 [− 0.45, 0.97]	0.74 [0.32, 0.88]	0.72 [0.27, 0.86]	0.63
Southern Guadalcanal (CMT_SG_)	14	− 0.09	− 0.09	0.01	0.0	0.0

Summary of regression and correlation statistics including 95% bootstrap confidence intervals (1000 bootstrap samples)

SOI showed a significant correlation at the 95% confidence (p < 0.05) level with malaria transmission in Guadalcanal only for the 21-year dataset (Table 1). NINO3.4 and mean sea level (MSL) in Honiara were found to be significantly correlated with the transmission for the 14-year dataset and in northern Guadalcanal (CMT_NG_) only (Tables 1, 2 and 4).

**Table 4:** 14-year southern Guadalcanal

Variable	p value	Adjusted R^2^	Coefficient	Intercept
Rainfall	0.14	0.10	− 0.1156	143.39
MSL	0.47	0.0	− 89.89	145.78
NINO3.4	0.77	0.0	0.0239	0.0082
SOI	0.72	0.0	− 0.0033	0.0215

Summary of linear regression results for data (confidence intervals not included as results were not significant)

The most important result of the stepwise regression was that only rainfall showed significance at the 95% confidence level (p < 0.05) indicating that the relationship between climate and malaria was effectively encapsulated by rainfall at Honiara; adding other climate variables to the regression did not provide a significant improvement to the model fit.

In southern Guadalcanal, none of the climate variables in the analysis was found to be significantly (p < 0.05) correlated with climate-associated malaria transmission (CMT_SG_) (Table 4). There was little correlation between climate-associated malaria transmission in Guadalcanal Province and southern Guadalcanal (R^2^_GP–SG_ = 0.20).

In contrast, comparison of the 21-year Guadalcanal Province and 14-year northern Guadalcanal model over the period 1998–2013 showed a close relationship between the two $\left( {{\text{R}}^{ 2}_{{{\text{GP}} - {\text{NG}}}} = 0. 90} \right)$ meaning that most of the variability in Guadalcanal malaria was adequately explained by the sub-provincial level data, i.e. northern Guadalcanal.

As expected from such a small dataset, R^2^ shrinkage was evident when LOOCV was performed, although both the 21-year Guadalcanal and 14-year northern Guadalcanal models performed reasonably well.

### Threshold determination for operational use

In the context of applying this model to a simple malaria early warning system, malaria transmission was separated into three-tiered categories: above normal, normal and below normal. Above normal was defined as years where CMT was in the upper tercile while below normal was defined as years where CMT was in the lower tercile. This is consistent with the methodology used by local climate services to define periods of high, medium or low rainfall in seasonal climate outlooks.

Using this definition, the threshold between upper and middle tercile rainfall (67th percentile) was 592 and 548 mm for the northern Guadalcanal and Guadalcanal Province datasets respectively, whilst the threshold between the lower and middle terciles (33rd percentile) was at 403 and 358 mm, respectively, over the OND period. The lower thresholds exhibited by the Guadalcanal dataset reflects the fact that the 1988–1999 period was significantly drier than the 2002–2013 period (remembering that the Guadalcanal Province dataset runs from 1988–2013 while the northern Guadalcanal dataset runs from 1998–2013).

**Fig. 9:**
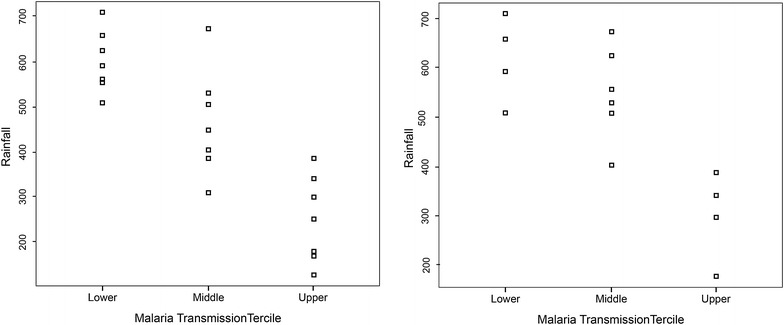
Rainfall threshold. Strip chart of rainfall (mm) linked with lower tercile, middle tercile and upper tercile of climate-associated malaria transmission for (**a**) 21-year Guadalcanal Province and (**b**) 14-year northern Guadalcanal models

To determine whether there was a statistically significant difference in rainfall associated with upper, middle and lower tercile malaria years, the Mann–Whitney U test [51] was applied to both the upper and lower terciles of CMT_NG_ and CMT_GP_. The test demonstrated that in both models the upper and lower sample groups (“events”) differ from the remainder of the data (“controls”) at the 95% level (p < 0.05). This can be seen in Fig. 9a, b which also shows that the upper and lower malaria tercile years are distinct. For northern Guadalcanal there is no overlap between the middle and the upper terciles. There is, however, significant overlap between the lower and middle tercile, meaning that the model is not as effective at distinguishing between below normal and normal malaria transmission years (Table 5).

**Table 5:** October–December rainfall and subsequent January–June malaria incidence

Model	Tercile	Events	Controls	U-score	p value
Guadalcanal (CMT_GP_)	Upper	7	14	95 [89, 98]	p = 0.0001
	Lower	7	14	91 [78, 98]	p = 0.0008
Northern Guadalcanal (CMT_NG_)	Upper	4	10	40 [40]	p = 0.0020
	Lower	4	10	33 [23, 40]	p = 0.0759

Summary of non-parametric test statistical test results with 95% bootstrap confidence intervals shown where applicable (1000 bootstrap samples)

## Discussion

### Implications of statistical relationships

The results obtained from both models suggest that higher total rainfall in the OND period is linked with a reduction in observed malaria cases in the subsequent January to June period. Conversely, a reduction in OND rainfall is linked to an increase in observed malaria cases in the subsequent January to June period. This relationship holds whether looking at northern Guadalcanal over a period of 14 years or the whole of Guadalcanal over 21 years. It is unclear whether this relationship would hold at very low rainfall (> 150 mm in OND) as occurrences of this are very rare, and none exist within the period for which malaria data are available. The fact that lower malaria incidence is typically observed following the Solomon Islands dry season between July and December suggests that *extremely* low rainfall may lead to a decrease in malaria transmission.

Much of the strong downward trend in malaria cases after 2005 is attributable to improved control measures over this period and is not a result of rainfall variation or other environmental impacts. These results reinforce the importance of maintaining and expanding malaria control efforts so that in the future, conditions more favourable to malaria transmission do not lead to corresponding rebounds in malaria case numbers.

It is believed that the influence of rainfall on malaria transmission is primarily a result of the effects that rainfall accumulations have on the hydrological environment in the Solomon Islands. However, a lack of long-term hydrological data makes confirming the veracity of this difficult.

Malaria transmission in southern Guadalcanal over the period 1998–2013 was not found to be significantly correlated with rainfall, NINO3.4 or SOI. Since the vast majority of the human population and malaria cases are in northern Guadalcanal, statistical relationships established at the provincial level are not a reliable indicator of the effects of climate on malaria transmission in the southern region. At present, there is insufficient local data available to confirm the nature of the relationship between climate and malaria in the southern region. This reinforces the importance of local climate data and its importance for understanding any relationship to malaria that exists on the southern coast of Guadalcanal.

### Links to known physical phenomena

**Fig. 10:**
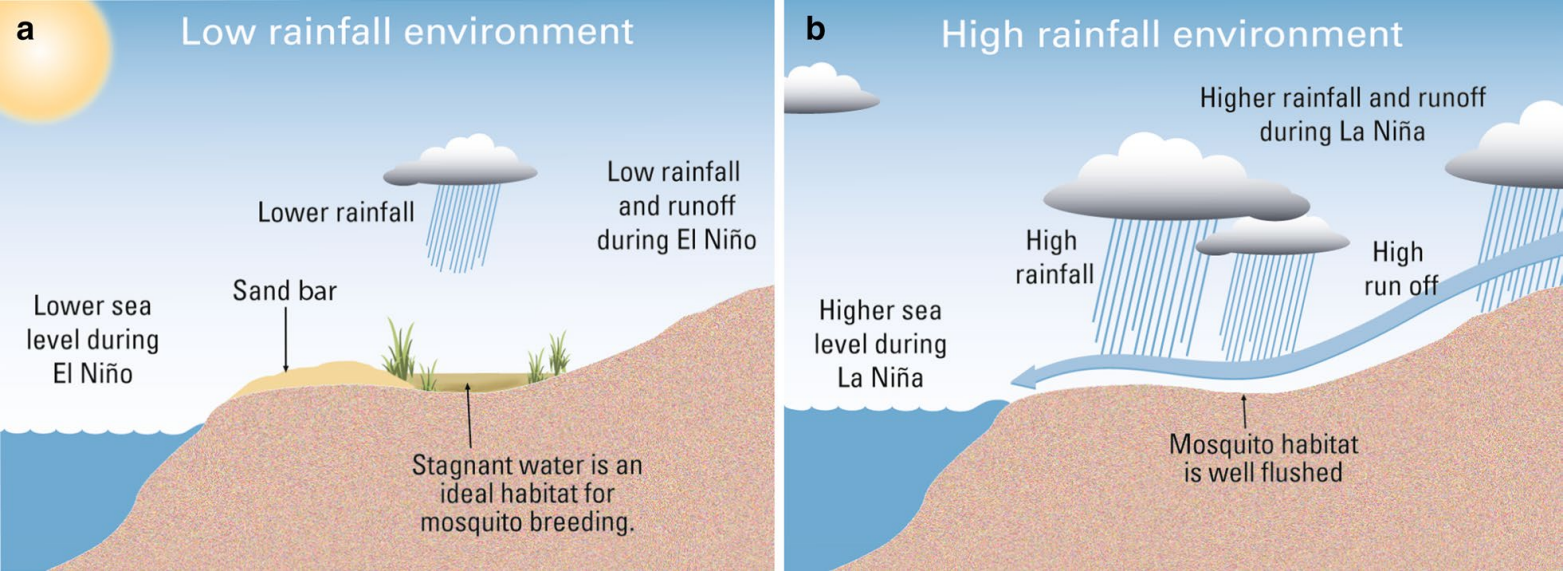
Sand ridge mechanism. **a** Sandbar formation in drier years creates ideal coastal habitats for *Anopheles farauti.* **b** Excess runoff in wetter than normal years tends to flush these habitats

The mechanism responsible for the negative relationship between rainfall and malaria transmission in northern Guadalcanal has not been conclusively established, but local studies point to several possible explanations [18, 30]. In northern Guadalcanal it has been observed that reduced rainfall and lower sea levels (both of which are linked to the El Niño phase of ENSO) cause sediment build-up across the mouths of estuaries, producing sandbars which block the outflow of these permanent water courses. These blockages create calm, stagnant pools of brackish water which provide an ideal habitat for the primary malaria vector, *Anopheles farauti* [30]. Drier years also result in lower-than-normal streamflows throughout river systems thereby increasing the chances of stagnant ponds being formed along the banks of these river systems. This in turn expands the availability and spatial extent of suitable habitats for *Anopheles farauti* (Fig. 10a).

In contrast, La Niña phases are associated with higher-than-normal rainfall and water levels which tends to reduce the brackishness of the coastal estuaries and swamps due to the increased volume of fresh water flowing through river systems. Higher streamflows also reduce the likelihood of sandbars forming across the mouths of estuaries, and increase the chances of sand bars being broken earlier. This in turn reduces the suitability of vector habitats along the water course, particularly near the river mouth. These factors reduce the spatial extent of *Anopheles farauti* preferred habitat, which in turn leads to reductions in vector populations and hence seasonal malaria transmission (Fig. 10b).

This hypothesis is reinforced by studies undertaken in northern Guadalcanal [18, 30], which have found that reductions in vector populations coincide with higher rainfall seasons, while the highest population of vectors is found during lower rainfall seasons. However, observations in neighbouring Vanuatu noted that increases in rainfall sometimes have the effect of opening up temporary suitable habitats, whilst drier than normal years make these transient habitats less viable [52].

Studies from other parts of the world indicate that increased wet season rainfall in already high rainfall regions leads to accumulations that flush vector breeding habitats, particularly where these breeding habitats are linked to permanent water courses [13–16]. This contrasts with drier regions, where vector species often rely on transient habitats which are created or expanded following significant rainwater accumulations [19–24].

It is hypothesized that the nature of the relationship between rainfall and malaria transmission is largely driven by the proportion of suitable vector habitats which are either permanent or transient in nature. In regions where permanent bodies of water courses are plentiful, vector breeding is likely to be centred on these permanent water bodies, and the relationship between malaria transmission and rainfall is more likely to be negative.

The broad coastal plain of northern Guadalcanal is unique in the Solomon Islands for being the only large area of low flat plains. This landscape increases the residence time of water in local rivers systems, resulting in more stable flows and water heights with less seasonal and annual variability. The result is that the majority of rivers in northern Guadalcanal are perennial, providing permanent vector habitats. In contrast, much of the rest of the Solomon Islands consist of rugged, smaller islands where the residence time of river systems is substantially less and more water courses are ephemeral or intermittent. This would indicate that the relationship between rainfall and malaria transmission is likely to be different from that observed on the northern, leeward side of Guadalcanal.

More extensive research in Temotu [31] and Santa Isabel [32] indicates that these areas exhibit quite different characteristics from those found in northern Guadalcanal. Preliminary research and discussions with local control authorities suggest areas of northern Makira and Central Province of the Solomon Islands possibly exhibit similar habitat characteristics to northern Guadalcanal. Entomological studies recently conducted in Central Province have investigated coastal larval habitats where water flow into the ocean was blocked by a sand mouth [53]. This deserves further investigation.

### Rainfall-based malaria early warning system for northern Guadalcanal

The statistical results of this study validate using OND rainfall as an indicator to provide early warning about the potential severity of the subsequent malaria season between January and June in northern Guadalcanal. However, the achievement of positive outcomes will require a close ongoing relationship between the SIMS and NVBDCP alongside the development and implementation of supporting tools, frameworks and capacity building activities.

In collaboration with the SIMS and NVBDCP, it was decided that a three-tier categorical warning system would be the most appropriate format for disseminating malaria alerts for the upcoming season. This meant that the relationships explored earlier had to be transformed into three discrete warning levels: below normal, normal and above normal. In climate, three category statistical forecasting systems are typically derived from the terciles of the historical climate dataset being used.

**Fig. 11:**
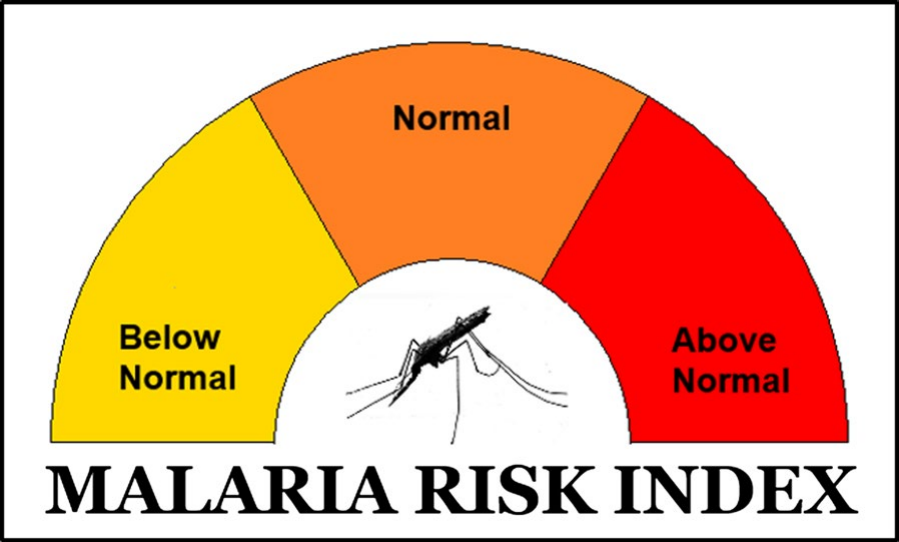
Simple malaria alert dial to be used by SIMS and NVBDCP for malaria early warning. SIMS provide customised rainfall outlook to NVBDCP malaria services with a dial representing the forecasted climate component of the transmission risk

An appropriate lower threshold below which the chance of malaria transmission would be higher was determined to be 350 mm of rainfall at Honiara in the OND period. Similarly, it was determined that 550 mm of rainfall in the OND period was an appropriate threshold above which the chance of enhanced malaria transmission would be lower. These thresholds, along with routine data collection from their rainfall monitoring network, will allow the SIMS to produce a rainfall-based malaria outlook, including a rainfall-based malaria alert dial (Fig. 11), for the NVBDCP covering the northern Guadalcanal region.

## Conclusion

This study attempts to quantify the relationship between climate and malaria in Guadalcanal on an inter-annual timescale. Strong relationships (R^2^ = 0.74, p < 0.05, n = 14) have been established between observed October–November–December (OND) rainfall in Honiara and subsequent January–June climate-associated malaria transmission (CMT) in northern Guadalcanal. The analysis shows a correlation between the OND rainfall and malaria transmission in the following January–June period: in years of below normal rainfall, the malaria incidence tends to be higher, while in years of above normal rainfall, malaria incidence tends to be lower.

These results support the hypothesis that changes in seasonal rainfall—often associated with the El Niño southern oscillation—have an effect on the likelihood of malaria transmission in subsequent malaria seasons. This relationship is believed to be linked to the specific hydrological characteristics of the northern Guadalcanal region and the habitat preferences of the primary vector species, *An. farauti*. Previous studies have shown that drier years promote the formation of sandbars at the mouths of permanent estuaries, which increase the availability of the stagnant brackish marshes, the preferred habitat of *An. farauti.* In wetter years, rainfall accumulations cause increased hydrological flows which result in these habitats being flushed. It is this mechanism that is believed to be primarily responsible for the strong links between rainfall and malaria transmission in northern Guadalcanal.

This study is now being used by the SIMS and the NVBDCP to provide the basis of a climate-based malaria early warning system with the support of COSPPac into a monthly customised outlook. It is currently available only for northern Guadalcanal. Since the beginning of the project, rainfall gauges have been installed by SIMS in locations chosen in consultation with NVBDCP around the Guadalcanal Island. The MalaClim rainfall-malaria model can then be updated when enough available rainfall and malaria data is collected at a finer scale. It is hoped that the warning system will help to better integrate climate information with the malaria monitoring and control processes already in place at the NVBDCP. This will provide useful information for evaluating historical control measures by accounting for the favourability of prevailing climate and also better targeting future interventions and awareness activities. It is hoped that this will help the NVBDCP to achieve the most efficient and effective malaria management outcomes with the limited resources available.

## Appendix: glossary of climate terms

### Anomaly

In climate science, it is usually a deviation of a variable from the average value at a specific place and time.

### Inter-tropical convergence zone (ITCZ)

The ITCZ is a belt of converging trade winds and convection that encircles the Earth near the equator. The convection produces increased cloudiness, frequent thunderstorms, and heavy rainfall within the zone.

### South Pacific convergence zone (SPCZ)

The SPCZ is a major spur of the ITCZ that extends from the Western Pacific Warm Pool from approximately 140°E near the equator to southeast towards French Polynesia and the Cook Islands at approximately 120°W at 30°S.

### Western Pacific Warm Pool

The Western Pacific Warm Pool is a body of water encompassing parts of the western tropical Pacific and extending from the central Pacific to the far eastern Indian Ocean. It is typically defined as the region enclosed by the 28.5 °C sea surface temperature (SST) isotherm, meaning that its precise size and shape varies throughout the year.

### El Niño-southern oscillation (ENSO)

ENSO is an irregular oscillatory climate phenomenon associated with variations in SSTs over the tropical central to eastern Pacific Ocean. The warming phase is known as El Niño and the cooling phase as La Niña. A neutral phase also exists when there is neither significant heating nor cooling in the tropical central Pacific.

### El Niño phase

El Niño is the negative phase of the El Niño-southern oscillation. El Niño events are indicated by sustained negative southern oscillation index (SOI) values. El Niño refers to the extensive warming of the tropical central to eastern Pacific that leads to a major shift in weather patterns across the Pacific. El Niño events are often accompanied by cooler than normal SSTs in the western Pacific.

### La Niña phase

La Niña is the positive phase of the El Niño-southern oscillation, sometimes referred to as the “opposite of El Niño”. La Niña events are indicated by sustained positive values of the southern oscillation index (SOI). La Niña refers to the extensive cooling of the tropical central to eastern Pacific. La Niña events are often accompanied by warmer than normal SSTs in the western Pacific.

### Neutral phase

ENSO Neutral is a state of near-normal SSTs in the central to eastern Pacific Ocean, that is, neither El Niño nor La Niña.

### NINO3.4

NINO3.4 is measure of sea surface temperature anomalies in the Central Pacific region bounded by 5°N–5°S and 170°W–120°W. NINO3.4 is used to give an indication of the development and intensity of El Niño or La Niña events.

### Southern oscillation index (SOI)

SOI gives an indication of the development and intensity of El Niño or La Niña events in the Pacific Ocean. The SOI is calculated using the pressure differences between Tahiti, French Polynesia and Darwin, Australia.

### Trade winds

The trade winds are the prevailing pattern of easterly surface winds found in the tropics. In each hemisphere the trade winds head towards the west and towards the equator, as wind direction is conventionally described in terms of where the wind is coming from. The convergence of these winds from north and south forms the ITCZ.
